# What Do They Do? Construction of a Team Leader Intervention Model

**DOI:** 10.3389/fpsyg.2021.552521

**Published:** 2021-09-29

**Authors:** Kjell Ledin, Peter Bengtsson, Tore Ärlemalm

**Affiliations:** Engineering Psychology, Luleå University of Technology, Luleå, Sweden

**Keywords:** group interventions, behaviour inventory, inductive approach, team leadership, disturbing situations, assessment

## Abstract

This research report aimed to present a team leader intervention model regarding when unexpected events arise in meetings. Onward, the model will form a starting point for the creation and validation of a team leader interventions inventory. Sixteen managers provided the empirical material for the construction of the model. The subjects proposed as many interventions as possible based on 10 different group meeting scenarios. In total, 327 interventions were proposed, which constituted the basis for a conceptual framework comprising six categories—Control, Inform, Initiate, Investigate, Support, and Avoid. Three of the categories correspond to classical leadership behaviours: the Control category to Authoritative Leadership and Task Behaviour and structure; the Support category to Democratic Leadership and Relationship Behaviour and consideration; and the Avoid category to Laissez-Faire Leadership, letting events pass without taking leadership. In addition, the conceptual framework includes three new categories in addition to the classical leadership theory. The Inform category is related to the controlling function. When the leader clarifies goals and how to achieve the goals, it is indirectly a controlling function. The Initiate category is related to launching procedural or distracting activities. Finally, the Investigate category is an almost necessary step ahead of the other categories. Before controlling, informing, initiating, supporting, or avoiding, the leader ought to investigate the causes of the disorder and then decide which intervention is most appropriate.

## Introduction

Personality tests and inventories measuring traits are standard procedures used to select personnel and staff development and training. When selecting candidates for managerial positions, there are often demands on acting in groups and handling different events/situations. The individual must comprehend that there is a repertoire of possible interventions available in each group meeting situation. An assumption in this study is that the individual who has a broad repertoire of interventions/behaviours has better opportunities to succeed in management positions that require group leadership skills than the individual relying on a one-sided pattern of interventions, no matter the situation. This assumption is in-line with Reunanen and Kaitonen (2016), who argue that leaders need different styles in their functional roles.

A trait is a descriptive term pertaining to personality that primarily concerns the measurable, consistent aspects of personality. Trait theory assumes that the ability to lead is acquired in childhood or inherited (Colbert et al., 2012). However, researchers have questioned the notion of inherited leadership, arguing that leadership skills do not have to be innate for a leader to perform a good and successful job (Heide et al., 2015).

Trait theory includes persisting cognitions, emotions, and behaviours that distinguish us as humans from each other (Funder, 1991). Trait theory has had a significant impact for many decades on personality theory and measurements, from Cattell's sixteen-factor model and Eysenck's three-factor model to the more recent Big Five personality traits or five-factor model (Eysenck, 1991). The five factors in the model are openness to experience, conscientiousness, extraversion, agreeableness, and neuroticism (McCrae and Costa Jr, 1987). The five-factor model, widely used for personality assessments, has been repeatedly verified in North America and Western Europe (Saucier et al., 2000).

The frequently administered personality assessments are valuable in various human resource management activities, but there are matters of opinion regarding utility. Several studies have made it clear that personality assessments based on trait theory only partially predict leadership success (Mumford et al., 2001). A critical discussion concerns how we best can understand the construct of personality in situations, based on enduring personality traits and changing social contexts, respectively (Bengtsson et al., 2019).

*Integrated leadership (Pauchant*, *2005**) is a management model where the focus is on people's perceived reality, external and internal reality, and individual and collective reality. Pauchant also mentions that many researchers want to replace the word “follower”, implying that the leader acts while the group follows and obeys. However, according to the integrated leadership model, the leader takes on an influential and developing role, and the group then consists of employees instead of followers*.

While trait theory heavily relies on de-contextualised traits to explain personality and social personality theory by Albert Bandura and Walter Mischel emphasise the role of environmental influences in personality (Bandura, 1999; Mischel, 2004). According to social personality theory, there are many aspects of self-valued or functional in different circumstances. Thus, social worlds actualise different aspects of the self; for example, a person can be extrovert and talkative in one social situation, but quite the opposite in another (Bengtsson et al., 2019). Identity formation is consequently characterised as an ongoing process and not fixed in time.

Given the social personality theory perspective concerning our highly conditional nature, personality assessments cast in non-conditional generalities cannot sufficiently capture individual psychosocial functioning in diverse task domains and through all situations. Consequently, personality assessments need to capture the contextualised and multifaceted nature of human functioning. Theories have emerged focusing on the psychological content of different situations regarding personality assessments and recruitment activities, e.g., the situational personality eight DIAMONDS taxonomy (Rauthmann, 2017).

Contemporary research argues for the construction and use of situational taxonomies to investigate the potential of features (Rauthmann, 2017). Situations, especially their psychological content and style characteristics, are diagnostic agents for detecting individual differences among job candidates, existing personnel, or meaningful or essential leaders for specific tasks, occupations, teams, work settings, or organisations.

The situational theory of Hersey and Blanchard (1993) means that “maturity” among employees determines which leadership style to use. If maturity of the subordinates is low, the task orientation of the leader should be high and the relationship orientation should be low. If maturity is average, the relationship orientation of the leader must be high and task orientation must be average. If the maturity of the employee is high, both the relation orientation and task orientation should be low.

Hersey and Blanchard emphasise that most people have a style that they prefer, but they claim that flexibility rather than consistency is a hallmark of effective leadership. The test design is a mapping of this consistency and flexibility sought, what leadership style prefers the individual usually, and how flexible the individual is to use other leadership styles.

According to Fielders Contingency model (Fielder, 1967), effective leadership behaviour is due to:

a) The motivation style of leader
  - The ones that motivate through the task corresponds to the categories Control and Inform, which means a task-oriented leadership.
  - The ones that motivate through relationships corresponds to support, which means leadership that is relation oriented.
b) The leader's control in the problem-solving situation
  - Loyalty in the group to the leader or the internal cohesion of the group.
  - The structure of the task, i.e., if the task is clear, structured, and there is only one correct solution. An option that the task is unstructured, unclear, and there is no “correct solution.” In the 10 situations, the starting point is the latter because the test then assumes more of the characteristics of the tested and thereby discriminates better between different individuals.
  - The position power of the leader, i.e., the power that a leader usually has in a meeting with a task to perform. In the instruction to the test, it is assumed that the power of a leader is the usual position power in a group who shall perform a task.

Fielder's Contingency Model further suggests that a task-oriented leader is more efficient in a situation with very low or very high control/structure. He explains the differences between different leaders as differences in target priorities. Some leaders prioritise establishing and maintaining good relationships, and other leaders prioritise performing/completing the task. Thus, we are back close to the classical leadership theory with authoritative/task-oriented and democratic/relationship-oriented leadership.

*Given the social personality theory perspective concerning our highly conditional nature, personality assessments cast in non-conditional generalities cannot sufficiently capture individual psychosocial functioning in diverse task domains and through all situations. Consequently, personality assessments need to capture the contextualised and multifaceted nature of human functioning. Theories have emerged focusing on the psychological content of different situations and how this can be used in personality assessments and recruitment activities, e.g., the situational personality eight DIAMONDS taxonomy (Rauthmann*, *2017**) who argues for support for the construction and use of situational taxonomies to investigate the potential of features: “We can use situations, especially their psychological content and style characteristics as diagnostic agents for detecting individual differences among job candidates, existing personnel, or leaders that are meaningful or important for specific tasks, occupations, teams, work settings or organisations.”*

The Eight DIAMONDS taxonomy contains the dimensions Duty (work and tasks), Intellect (processing and problem-solving), Adversity (criticism and domineering), Mating (sex and romance), Positivity (fun and playfulness), Negativity (stress and frustration), Deception (mistrust and sabotage), and Sociality (relations and cooperation). A particular DIAMONDS dimension Duty is supposed to be salient to activate diagnostically interesting individual differences in trait-relevant behaviour in work and tasks. This dimension Duty is a relevant area for the construction of situations in this test.

According to the situational leadership theory, effectiveness and success of a leader depend more on the flexibility and adaptation of the leader to the requirements of different situations than by personal traits. Situation tests where participants face realistic and challenging real-life scenarios, each accompanied by several possible response options, and the participants take a position can be an alternative way of mapping the existing management requirements. For several years, the United States and England have accumulated support for this approach. Situation tests have shown a higher correlation with managerial performance than existing aptitude and personality tests (Howard and Choi, 2000).

*This work aims to construct a model that describes the group interventions of leaders based on how existing leaders describe them*.

This model had an inductive approach as a starting point for constructing and validating an inventory that measures interventions of leaders in situations where unexpected events arise.

## Method

### Participants

*Sixteen managers participated in the study-*−*12 men and 4 women, between 30 and 50 years of age, and with managerial experience from 1 to 10 years. All worked in the same business and service-producing countywide organisation in Sweden. An essential part of their managerial work was to lead meetings with employees concerning business focus and standard policies. The participants had signed up for a leadership development program that evaluated their managerial potential through various tests*.

### Materials and Instructions

*The material comprised examples depicting 10 different meeting scenarios in a working group based on Jones and Pfeiffer (**1975**). The selection of scenarios followed a purposive sampling approach (Silverman*, *2000**). Each scenario described an ordinary event that acted as a disturbance while allowing several leadership interventions regarding teamwork and goal achievement*.

*All 10 scenarios included the following information:*
  - *It is YOU who is the leader/chairman of the group*.
  - *The goal of the group's work is clearly defined*.
  - *The group size is 8–14 employees*.
  - *All employees have some skills that are important for the group's work*.
*The participants got the following instructions:*
  - *Read the description of the situation*.
  - *Think about which different interventions should be possible to do as a leader as you see and write down all of them*.

### Ten Scenarios

The 10 scenarios that had to be addressed by the participant were as follow:

- To start. You are the leader/chairman of this group that meets today. Everyone is in place when you get into the room and sit down. You present yourself, and the employees present themselves in turn. Then everyone turns to you expectantly. It is quiet. What are you doing now?
- Another topic. The group uses most of the time to talk about things that only indirectly affect the work of the group. No one seems to be content with the discussion, and it seems that this discussion would continue throughout the meeting. What are you doing now?
- Too late arrival. It is the third meeting. One of the employees is entering the meeting one quarter after the start. Although the employee did so at the previous meetings, nobody says anything. What are you doing now?
- A dominating group member. At one of the previous meetings, one of the employees dominated the discussion. Long monologues on behalf of the employee slow down the development of meaningful discussions. It is now in the middle of the meeting. The employee has once again talked for a more extended period. What are you doing now?
- One member is crying. It is in the middle of the meeting. A colleague who has been silent during the first half of the meeting makes an effort to gain control and starts to cry. Nobody says anything. What are you doing now?
- A silent member. One of the employees has said very little in the meeting so far, although he/she seems to keep up with everything that happens. It is now at the end of the meeting, and the other employees begin to ask about the silence of the employee, but he/she does not answer the questions, and the group seems unsure how to act. What are you doing now?
- The negative group. The meeting is characterised by irritation and negativism. Each time someone has a proposal; it is questioned and considered by somebody as not feasible. No one seems satisfied with anything. The committed atmosphere of the last meeting is wholly lost. What are you doing now?
- Subgroups develop. The group has spent a great deal of time thoroughly discussing the prerequisites for an investment decision. Some employees start a conversation that interferes with the discussion. They show no signs of ending their conversation. What are you doing now?
- The group attacks. After the group has spent half of the time talking about things that only indirectly affect the work, it turns to you and accuses you of being detached and quiet. What are you doing now?
- A member with difficulties. During a meeting, one of the employees takes up a personal problem. The employee is out of balance, hoping for one to the other but always turning back to the same boring fact. The employee has constantly looked at you and ignored the rest of the group. After completing an entry, he/she asks you directly about your opinion. What are you doing now?

### Procedure

The 16 managers did an entire test battery to chart their leadership development potential. At the end of the test day, the managers responded to the questionnaire with the 10 meeting scenarios described above, one scenario per page. The instruction was that they should come up with and write down as many interventions as they could think of for each of the scenarios. In the scenarios, the participants were the chairpersons or leaders of an imaginary working group of 8–14 members. All of the members had some competence that was important for the group's work and, consequently, the organisation. The goal and purpose of the imaginary group's work were clearly defined, and the group had already met before. The participants received information that their written answers were only for research purposes, submitted anonymously, and once compiled would be deleted.

### Data Processing

The proposed interventions constituted the basis for categories that comprised interventions as similar as possible within the categories and as different as possible between the categories. A conceptual six-frame reference was constructed based on this coding. The conceptual reference frame and underlying data were further analysed by gaps in focus points and relationships according to “Repacking and aggregating data-searching for relationships.” An interview with an experienced executive search consultant mapped out common leadership behaviours in meeting situations. The results were analysed and compared to the conceptual frame of reference through cross-case analysis (Miles and Huberman, 1994).

## Results

The participants proposed a total of 355 interventions in the 10 scenarios. Of these, 28 proposals were identical. Resided 327 different proposals for interventions. The proposals spread pretty evenly over the 10 situations with 42 proposals for Situation Nos. 1 and 2 (to start, another topic) and at least 22 Suggestions for Situation No. 9 (A member cries). The variance in the total number of answers between the chief aspirants ranged from no more than 39 to at least 13 proposals. Based on the collected data, analyses was done by: emphases, gaps, other dimensions, and relationships, according to repacking and aggregating data search for relations (Miles and Huberman, 1994).

### Emphases

Refers to essential starting points for the analysis/coding

- The group meeting situations are stimulus examples to attract a different group leader behaviour.
- The overall repertoire of group leadership behaviour shall be the basis for the development of a psychological test that can be complementary to a personality test.

### Gaps

Refers to data that falls outside of the analysis performed, i.e., the proposals that fall outside the six categories of the conceptual reference frame. Here are examples of other methods for mapping these proposals:

- Inward interventions against myself as a leader—Interview.
- Show your feelings—Interview. Observation.
- Using their body language/strength in performance—Observation.
- View/act early.
- Observation—. —Customise their language to the recipient/circumstances.

### Other Dimensions

Refers to data that are difficult to handle within the existing reference frame.

- Own emotions/inward interventions.

### Relationships

Refers to how the categories are related to each other

The qualitative analysis categorised six factors with the following content:

1. Control: Acts as a leader by ruling on its authority as a leader to be the one who determines or rules. Acting on its plan, leader, interrupting, pointing out, and addresses individuals.
2. Inform/clarify: Acts as a leader by informing about the goal of the meeting and clarifying the importance/importance of achieving that goal (actually targeting), explains, shows, reminds, values, and focuses.
3. Initiate: Acting as a leader by initiating/launching procedural or other activities.
4. Support: Acts as a leader by supporting, accepting, and taking into consideration the participants. Trying to get involved and have a dialogue to find shared solutions. Comforts, encourages, and accepts feelings to come true.
5. Investigate: Acts as a leader by investigating/analysing. Discusses the event with the employees, finds reasons, asks questions for explanations, and talks with individual employees directly in the group.
6. Avoid: Acts as a leader by waiting and let the event pass. Keep silent to give another chance to be active, delegate the procedural issues of the meeting. Avoid inhibiting the group by taking the lead.

**Figure 1:**
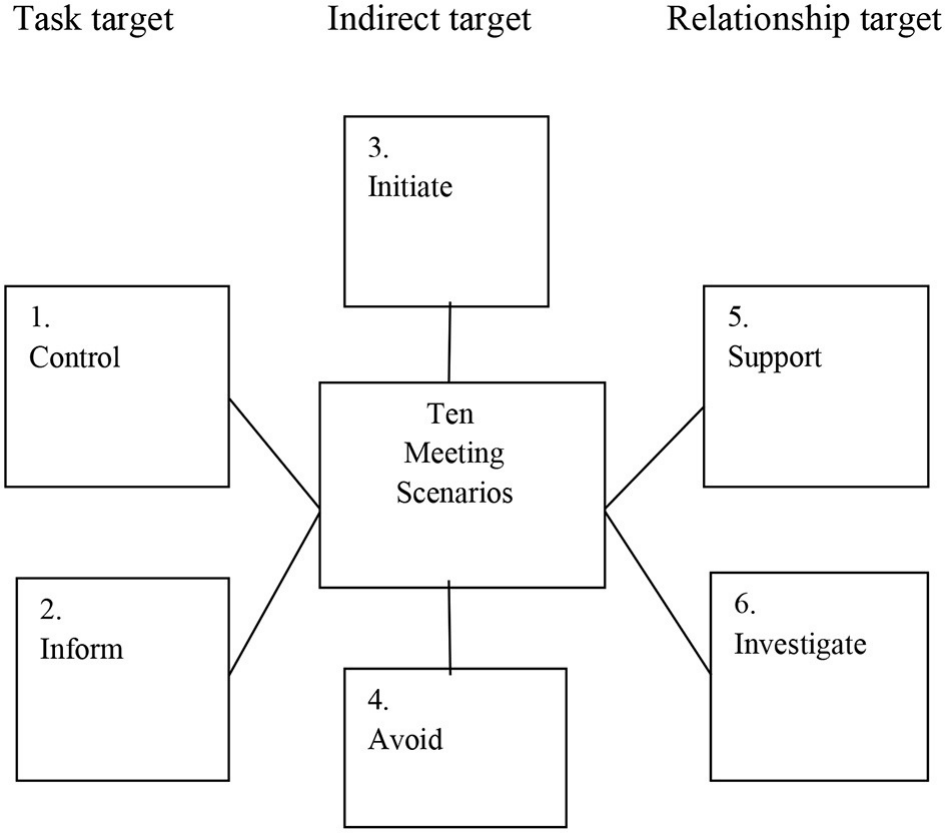
Conceptual frame for leadership interventions in meeting situations.

The outcome of the analysis and coding was a conceptual frame of reference with six categories (Figure 1).

Figure 1 shows six different leadership behaviour targets in meeting situations. Categories 1 and 2 have a task/structure target by control and information about the goal and task. Categories 3 and 4 have an indirect target initiating other activities or avoiding the problems so that the situation can calm down or letting the group or the employees solve the problem themselves. Categories 5 and 6 have a relationship target and deal with supporting groups or individuals or inviting them to investigate the problem and discuss a common solution.

To identify in a different way which leadership behaviours are the most important, the analysis included a personal interview with open questions with an experienced executive recruiter. According to Huberman and Miles, the interrogation response was analysed and compared to the conceptual reference frame by cross-case data analysis. Coding of the interview response showed that all six categories of the model were represented with the highest number of responses to Inform, Support, Control, and the lowest number of responses for Await, Investigate, and Initiate.

Two external independent assessors (one teacher and one postgraduate student) sorted independently from each other regardless of the earlier categorisation of the 327 proposals into the six established categories plus one category other. The consistency in categorisation with the two external assessors was highest in Investigate and lowest in Initiate.

## Discussion

The qualitative analysis of the proposals for leadership interventions showed a correlation with the classical leadership theory and behavioural theories as a product of what leaders do in their roles. The Control category corresponds to Authoritative Leadership and Task Behaviour (Structure). The Support category corresponds to Democracy Leadership Studies (Hempill, 1950), Democratic Leadership, and Relationship Behaviour (Consideration). The Await category corresponds to the Laissez-Faire Leadership (Smither, 1994). In the qualitative analysis, three new categories completed the classical leadership theory.

The Inform category appears to be related to the controlling function. However, it also relates to a selling function (Kolb et al., 2016). When the leader clarifies goals and paths to the goal, instead of controlling based on his superior function, it is indirectly a controlling function and an informing and selling function. The relationship between the two categories, Control and Inform, will be investigated in the ongoing work.

The Investigate category has emerged as a result of the inductive research method and is evident. Before a leader manages, supports, or waits, an investigation should determine which action is the most appropriate. The Initiate category shows behaviours that are neither direct task-oriented nor relationship-oriented.

A match exists with the Contingency model (Fielder, 1967). Effective leadership behaviour is due to the motivation style of the leader. Either the leader motivates through the task that corresponds to the categories, Control and Inform, or the leader motivates through relationship, which here corresponds to Support. The word support is of upbeat signification in leadership and also in the latest research (Meneghel et al., 2016).

*The Wait category is a neutral intervention that can be both bad and good. It can mean silence, uncertainty, and a conscious stance to wait for the activity of the group or judge that the issue is not sufficient for intervention. According to Wheelan (**2016**), effective teams need different leadership types depending on what phase the group is in, and that leadership needs to be flexible depending on the situation*.

In the instruction, the leader gives control in the problem-solving situations according to Fielder's Contingency Model. According to the test instruction, the participants had the usual position of power that a leader has in a group that shall perform a task. The group has the presumptions to be loyal to the leader because they have a common goal, they have had some meetings earlier, and the team of 8–14 employees has all competence that is important for the work. Nevertheless, the disturbing 10 events that occur are each unexpected, unstructured, and unclear so that the test measures the characteristics of the tested and thereby discriminates between different individuals. Fielder's Contingency Model further suggests that a task-oriented leader is more efficient in a situation with very low or very high control and structure. The test design does not assume such direct extremes and hence is not taken into account. No situations are considered that require urgent intervention or where failure to act swiftly has serious consequences, such as accidents. The 10 situations only cover those where leaders and groups work under “normal” circumstances and meeting-like forms. Fielder explains the differences between different leaders as differences in target priorities. Some leaders prioritise establishing and maintaining a good relationship, while other leaders prioritise performing and completing the task. Then we are back close to the classical leadership theory with task-oriented and relation-oriented leadership.

Hersey and Blanchard (1993) situational theory means that “maturity” among employees determines which leadership style to use. If the test instruction describes all employees as “having competence,” they can be comprehended mature and be treated equally with task-oriented and relationship-oriented leadership.

The 10 selected scenarios were not representative of all different meeting situations that may occur in workgroups. In this context, the choice was solely to attract various proposals for leadership interventions. The 327 proposals for intervention constituted the basis for six categories. A seventh category, Other, contained suggestions that could not be attributed to the other six categories. These other proposals were primarily about interventions directed at the person as a meeting leader, against aspects, such as their feelings, thoughts, and body language. Since the aim was to collect proposals that describe a leader's way of intervening in group meetings, these “inward” interventions are not relevant to the present research and development work.

The additional categorisation of the 327 proposals made with two independent assessors of the six established categories plus one category Other showed an acceptable level of inter-estimate reliability in five categories and lower inter-estimate reliability in the Initial and the Other categories. Probably the inclusion of the category Other in the assessment process lowered the overall inter-assessment reliability. The two assessors interpreted several of the proposals in this category in some of the six other categories. Several of the 327 proposals from the participants were also not so well-formulated and were not easy to interpret to their content, which also lowered the overall inter-estimate reliability.

In the continued development work, the Initiate category representing active interactions that were difficult to categorise directly to the content constitutes an own category. However, the Other category does disappear

*The following steps aim to revise and develop the model to a prototype for team leader assessment and develop it to the first version of a completed intervention inventory, test–retest, item analysis, variable correlation, factor analysis, and validation against personality test or inventories. The best practise guide for developing leadership scale, according to the paper of Reunanen and Kaitonen (**2016**), is adapted from DeVellis (**2016**) elements for broad-scale development over four key elements: generating theory, item development, content adequacy, and empirical evaluation. These elements will be followed in developing this behaviour inventory and in a later step by completing this inventory by the leaders thinking and feeling when unexpected events arise in group meetings*.

## Conclusions

Our findings suggest that there are connexions with both the classical leadership theory and the two-factor theory. The leader makes a task-oriented or a relation-oriented intervention when disturbing events arise. As a supplement to these theories, the investigative intervention focuses on what causes disorder at a meeting. The findings also suggest that the task-oriented intervention comprises a direct controlling intervention and an informative intervention that is more indirectly controlling. We conclude that the results obtained provide a point of departure for a situation-based intervention inventory that, after development and validation, can be complemented by psychological tests based on the Trait theory and the Big Five model.

A majority of the tests used today in manager selection measure personal characteristics and traits. The present work aims to develop an inventory of leadership behaviour in disturbing meeting situations. An inventory that can complement existing test batteries when selecting managers and as a basis for the development or training of existing managers.

## Data Availability Statement

The raw data supporting the conclusions of this article will be made available by the authors, without undue reservation.

## Ethics Statement

Ethical review and approval was not required for the study on human participants in accordance with the local legislation and institutional requirements. The patients/participants provided their written informed consent to participate in this study.

## Author Contributions

All authors listed have made a substantial, direct and intellectual contribution to the work, and approved it for publication.

## Conflict of Interest

The authors declare that the research was conducted in the absence of any commercial or financial relationships that could be construed as a potential conflict of interest.

## Publisher's Note

All claims expressed in this article are solely those of the authors and do not necessarily represent those of their affiliated organizations, or those of the publisher, the editors and the reviewers. Any product that may be evaluated in this article, or claim that may be made by its manufacturer, is not guaranteed or endorsed by the publisher.
